# Gastrointestinal metastasis from primary sarcomatoid carcinoma of the lung: a case report and review of the literature

**DOI:** 10.1186/s12957-015-0599-1

**Published:** 2015-05-08

**Authors:** Chun-Hsien Chen, Wei-Ming Chen, Shui-Yi Tung, Cheng-Shyong Wu, Wei-Lin Tong, Kam-Fai Lee, Kuo-Liang Wei

**Affiliations:** Division of Gastroenterology and Hepatology, Department of Internal Medicine, Chang Gung Memorial Hospital, 6 Section West, Chia-Po Road, Putz City, Chia-Yi 613 Taiwan; Graduate Institute of Clinical Medical Sciences, College of Medicine, Chang Gung University, Taoyuan, Taiwan; College of Medicine, Chang Gung University, Taoyuan, Taiwan; Department of Anatomic Pathology, Chang Gung Memorial Hospital, 6 Section West, Chia-Po Road, Putz City, Chia-Yi 613 Taiwan; Current address: Chun-Hsien Chen, Department of Gastroenterology and Hepatology, Chang Gung Memorial Hospital, 6 Section West, Chia-Po Road, Putz City, Chia-Yi 613 Taiwan

**Keywords:** Gastrointestinal metastasis, Lung cancer, Sarcomatoid carcinoma

## Abstract

Gastrointestinal metastases in lung cancer are extremely rare. The report presents a rare case of primary lung sarcomatoid carcinoma with both gastric and colonic metastases, and reviews the literature about endoscopic presentation of colonic metastases.

## Background

Primary malignancies with gastrointestinal metastasis are quite uncommon. In lung cancer, about half of cases have distant metastases at initial diagnosis. The mostly involved sites are the brain, liver, adrenal glands, and bone. The reported gastrointestinal metastasis is 5% to 14% at autopsy studies [1-4]. On the other hand, the clinical prevalence of symptomatic gastrointestinal metastases is rare, about 0.4% to 1.7% in larger series [5,6]. Some authors suggested that gastrointestinal metastasis from lung cancer may be underdiagnosed in the cases, whose gastrointestinal symptoms are obscure or just regarded as part of generalized metastatic disease [7,8]. This report presents a rare case of primary lung sarcomatoid carcinoma with both gastric and colonic metastases and reviews the literature about endoscopic presentation of colonic metastases.

## Case presentation

**Figure 1:**
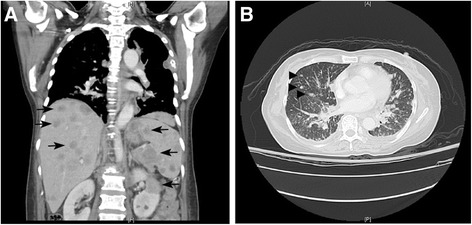
Chest and abdominal computed tomography (CT) of the patient. **(A)** Multiple liver, spleen, and left kidney metastases (arrow). **(B)** Lung nodules favor metastases (arrowhead).

**Figure 2:**
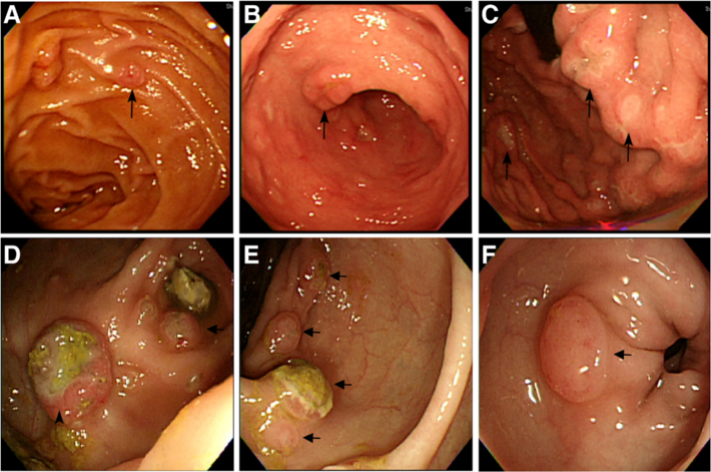
Endoscopic studies of the patient. **(A)** Duodenum. **(B)** Gastric antrum. **(C)** Gastric fundus and cardia. **(D)** Cecum. **(E)** Descending colon. **(F)** Sigmoid. EGD revealed multiple polypoid-like lesions with superficial erosion or ulcer (long arrow). Colonoscopy revealed one 1-cm submucosal tumor with central ulcer at cecum (arrowhead) and multiple nodules with variant size through the whole colon (short arrow).

**Figure 3:**
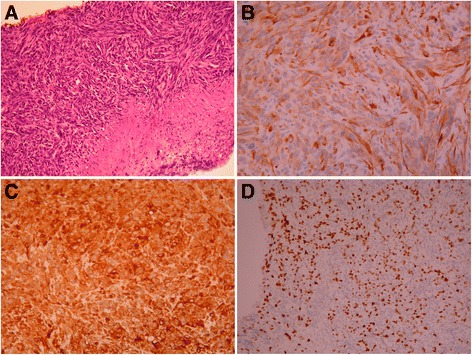
Microscopic appearance of the gastric lesion. **(A)** H & E, ×40. **(B)** Cytokeratin AE1/AE3 (CK AE1/AE3), ×200. **(C)** Vimentin, ×200. **(D)** Thyroid transcription factor-1 (TTF-1), ×200. The endoscopic gastric biopsy demonstrates sarcomatoid carcinoma. The carcinoma cells are positive for CK AE1/AE3, Vimentin, and TTF-1.

**Figure 4:**
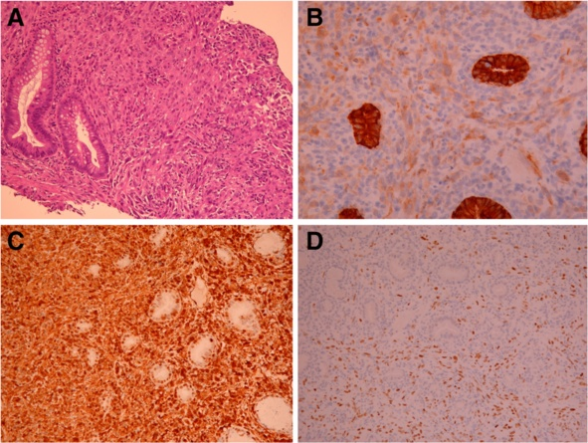
Microscopic appearance of the colonic lesion. **(A)** H & E, ×40. **(B)** Cytokeratin AE1/AE3 (CK AE1/AE3), ×200. **(C)** Vimentin, ×200. **(D)** Thyroid transcription factor-1 (TTF-1), ×200. The endoscopic colonic biopsy demonstrates sarcomatoid carcinoma. The carcinoma cells are positive for CK AE1/AE3, Vimentin, and TTF-1.

On June 25, 2014, a 59-year-old woman visited our gastrointestinal outpatient department because of chest tightness, abdominal pain, poor appetite, and body weight loss for 1 month. Multiple liver tumors were detected at other hospital. She denied smoking, alcoholism, or significant medical history before. Laboratory data also showed no evidence of past or persistent hepatitis B virus or hepatitis C virus infection. To survey probable malignant disease with liver metastasis, X-ray, abdominal echo, esophagogastroduodenoscopy (EGD), colonoscopy, and other examinations were arranged. Her chest X-rays showed multiple tumor shadows in both lung fields. Her abdominal echo showed liver cirrhosis, multiple liver and splenic tumors, and one 2.46-cm pancreatic head tumor. Chest and abdominal computed tomography (CT) revealed nodules and masses of variant sizes in the lung, liver, spleen, left kidney, and lymph nodes (Figure 1). For definite diagnosis of the disease, biopsy to liver tumor was performed on June 26, 2014. Histologic study showed pleomorphic, bizarre, and spindle cells. Immunohistochemically, the tumor cells were positive for cytokeratin AE1/AE3, Vimentin, and thyroid transcription factor-1 (TTF-1), while they were negative for caudal-related homeobox 2 (CDX2), cytokeratins 20 (CK20), hepatocyte paraffin-1 (HepPar-1), glypican-3 (GPC3), and S100 calcium-binding protein P (S100P). EGD showed gastric and duodenal polypoid-like lesions with central ulcer (Figure 2A,B,C). Colonoscopy showed multiple nodules and ulcerative polyps through the whole colon (Figures 2D,E). Bone scan demonstrated multiple bone metastases. Biopsies of the stomach (Figure 3), colon (Figure 4), and right rib displayed the same results as those for liver tumors. Bone marrow study also showed malignant cells involvement. Based on the tumor node metastasis (TNM) staging system, this sarcomatoid carcinoma, favor lung origin (TTF-1 positive), with bone, bone marrow, liver, duodenum, and colon metastases, was classified as stage IVB (pTxN2bM1b). The patient chose to receive supportive treatment for her malignant disease. At 1 month after initial diagnosis, she died because of severe sepsis.

### Discussion

The small intestine is the most common gastrointestinal metastatic site of lung cancer, with incidence rate of 4.6% to 10.7% at autopsy studies [1-4]. For the stomach and large intestine, the metastatic rates are 5.1% and 4.5%, respectively [6]. When small intestine involvement is noted, other concurrent metastatic sites should be considered [1,3,4]. McNeill and colleagues had reported an average of 4.8 metastatic sites in such cases [1]. Small intestine metastasis is also regarded as a poor prognostic indicator of lung cancer, with a mean survival of 2.3 to 4 months after diagnosis [6,7]. The causes include advanced diagnostic staging, high potential of metastatic disease, and poor general condition [3,7]. In our case, the sites of extra-gastrointestinal metastasis were three (liver, bone, and bone marrow), and the TNM staging was IVB (pTxN2bM1b). The patient only survived 1 month after initial diagnosis.

Most gastrointestinal metastatic lesions are silent, but they do cause symptoms when they become larger. Metastatic lesion may cause bulky tumor obstruction, bowel wall necrosis, bleeding, and most disastrously, perforation. The perforation is often spontaneous, but perforation after chemotherapy, maybe due to rapid tumor necrosis, had been ever reported [9]. When lung cancer had symptomatic intestinal metastasis, Di and colleagues had reported that the rates of perforation, obstruction, and gastrointestinal bleeding were 46%, 35%, and 14%, respectively [7]. In our case, the patient only had nonspecific abdominal pain. The duodenal and colonic metastatic masses did not cause obstruction, bleeding, or perforation, clinically. The endoscopic examinations were arranged to investigate the primary site of liver metastatic tumors at the very first time.

The incidence of gastrointestinal metastases disclosed by endoscopies is not common. Wei and colleagues had stated that the rate was 1 upper gastrointestinal metastasis per 3,847 panendoscopies and 1 lower gastrointestinal metastasis per 1,871 colonoscopies [10]. The endoscopic appearances of gastric metastatic lesions are variant and not specific for diagnosis. Submucosal tumor is the most presenting picture and account for 51% cases in one study [11]. When the lesions become larger, central erosion or ulcer may occur because of diminishing blood supply. Sometimes, the metastatic lesions may mimic primary gastric malignancies. There are three main morphologic types of metastatic lesion: (1) single or multiple nodules with variant size, with tip ulceration; (2) submucosal tumor with central ulcer, defined as ‘volcano-like’ lesion; and (3) raised lesion without central ulcer. Rarely, the lesion appears as polyp or raised plaques [11,12]. Due to vague symptoms, the endoscopic diagnoses of lung cancer with colonic metastasis are reported sporadically. Reviewing the literature from 2004 to 2013, 20 cases of lung cancer with colonic metastasis were found and 13 cases had undergone colonoscopy [6,13-28]. The results were summarized (Table 1). The most colonoscopic finding was submucosal tumor or polypoid-like lesion with or without central ulceration. Few cases had stenosis or obstruction. One case reported pancolitis [28], and one case failed to have an abnormal finding on colonoscopy despite a colonic metastasis disclosed by CT [27]. Almost the entire colon can be involved. In general, the endoscopic presentations of colonic metastases are similar to the morphologic patterns of gastric metastatic lesion. In our case, almost all morphologic types can be found. Both EGD and colonoscopy showed multiple polypoid lesions with central ulceration, similar to volcano-like lesions in previous studies. Additionally, there are multiple nodules with variant sizes through the whole colon. No luminal obstruction or gastrointestinal bleeding was found at our case.

**Table 1:** **Endoscopic features of lung cancer with colonic metastases**

**Author**	**Time**	**Numbers**	**Symptoms**	**Sites**	**Colonoscopy (lesion appearance)**	**Pathology**
Gonzalez Tallona AI [13]	2013	1	Rectorrhagia	Rectum	1. Lesion with button-like appearance, raised, smooth edges, and ulcerated in the center	NSCLC
					2. Polyp	
Sakai H [14]	2012	1	Abdominal pain	S-colon	Bulky disease with stricture	SqCC
Hsing CT [15]	2012	1	Abdominal pain	A-colon and D-colon	1. A 1-cm lesion at the proximal descending colon which looked like a submucosal tumor with mucosal bridge	Adenocarcinoma
					2. Ulceroinfiltrating lesion with spontaneous bleeding in the proximal ascending colon	
Cedres S [16]	2012	1	No symptoms (annual health exam)	Rectum	A 3-cm lesion with ulceration	SqCC
Huang YM [17]	2012	1	Abdominal pain	T-colon	A 1-cm tumor with central ulceration	Adenocarcinoma
Xue XY [18]	2012	1	No symptoms	A-colon	A 2-cm tumor with central ulceration	Adenocarcinoma
Fujiwara A [19]	2011	4	Abdominal mass	Colon	Nil	NSCLC
			Melena	Colon		
			Melena	Rectum		
			No symptoms	Colon		
Weng MW [20]	2010	1	Obstruction	Colon	Bulky disease with obstruction	Adenocarcinoma
Ahn SE [21]	2009	1	No symptoms	Cecum	Multiple cecal polypoid masses	Adenocarcinoma
Hirasaki S [22]	2008	1	No symptoms (positive fecal blood test)	D-colon	A 4-cm tumor with central ulceration	SqCC
Ma XT [23]	2008	1	Frequent loose stool	Cecum	Nil	SqCC
Goh BK [24]	2007	1	Abdominal pain	Cecum	Nil	Large cell carcinoma
Yang CJ [6]	2006	1	Bloody stool	Cecum	Diagnosis by colonoscopy, but no picture in the literature	Small cell carcinoma
Stinchcombe TE [25]	2006	1	No symptoms	A-colon	A 2-cm tumor with central ulceration	SqCC
Habesoglu MA [26]	2005	1	Abdominal pain	Colon	Nil	SqCC
Miyazaki K [27]	2005	1	Abdominal pain	Appendix	CT showed right pelvic mass, but colonoscopy failure to find obstruction or mucosal abnormalities in the ileocecal region	Adenocarcinoma
John AK [28]	2004	1	Diarrhea	Colon	1. Pancolitis and hemorrhagic inflammation	Undifferentiated large cell carcinoma
					2. Polypoidal lesions	

A-colon, ascending colon; D-colon, descending colon; NSCLC, non-small cell lung cancer; SqCC, squamous cell carcinoma; T-colon, transverse colon.

Due to no peculiar feature, it is difficult to diagnose the origin of gastrointestinal tumor by endoscopic pictures only. Additionally, synchronous lung, gastric, and colon cancers are not infrequent. Kurishima and colleagues had reported that the incidence rate of synchronous gastric cancer in patients with lung cancer was 3.2% [29]. Thus, biopsy is mandatory to confirm the diagnosis. All types of lung cancer can result in gastrointestinal metastasis [6,8,13-28]. TTF-1 is most expressed in lung and thyroid tumors and occasionally in tumors of the liver, colon, ovary, uterus, urinary bladder, and breast [30]. In lung cancers, Jerome and colleagues had reported that the rates of positive TTF-1 expression in adenocarcinoma, small cell carcinoma, and nonmucinous bronchio-alveolar carcinoma were 90%, 80%, and 100%, respectively. In contrast, the expression in squamous cell carcinoma and mucinous bronchio-alveolar carcinoma was often negative [31]. In metastatic cancers, TTF-1 is a useful marker to document the pulmonary origin if a thyroid or other less common origin had been excluded. Immunostaining with TTF-1, CDX2, CK7, and CK20 is helpful to distinguish primary gastrointestinal carcinoma from metastasis of lung carcinoma. Positive TTF-1 and CK7 staining suggested pulmonary origin, while negative CDX2 and CK20 staining can exclude the possibility of gastrointestinal origin [8]. Besides, negative HepPar-1 and GPC3 staining can exclude the possibility of liver origin, while negative S100P staining can exclude the possibility of pancreatic origin [32-34]. In our case, the pathologic findings of the liver, stomach, colon, and right rib were consistent, in which there was metastatic involvement from a primary sarcomatoid carcinoma of the lung.

Pulmonary sarcomatoid carcinoma is rare, accounting for 0.3% of all invasive lung malignancies. The tumor frequently presents as a solitary mass, located peripherally with a predilection for the upper lobes, large size with a mean diameter of 5 cm, and occasionally marked central necrosis with cavity formation. The average age at diagnosis is about 65 years, with a male and smoker predominance [35-38]. In our case, the patient’s clinical presentation was quite different from previous studies. She had multiple lung tumors rather than a single solid mass, and the tumor size was smaller without tumor necrosis or cavity formation. What is more, the patient denied any history of smoking.

## Conclusions

This report presents a rare case of primary lung sarcomatoid carcinoma with both gastric and colonic metastases. Endoscopy with histological examination is a way of identifying metastatic tumors of gastrointestinal tracts.

## Consent

Written informed consent was obtained from the patient for the publication of this case report and accompanying images. A copy of the written consent is available for review by the editor-in-chief of this journal.

## Abbreviations

CDX2: caudal-related homeobox 2
CK: cytokeratins
CT: computed tomography
EGD: esophagogastroduodenoscopy
GPC3: glypican-3
H & E: hematoxylin and eosin
HepPar-1: hepatocyte paraffin-1
S100P: S100 calcium-binding protein P
TNM staging: tumor node metastasis staging
TTF-1: Thyroid transcription factor-1
